# Much ado about flu: A mixed methods study of parental perceptions, trust and information seeking in a pandemic

**DOI:** 10.1111/irv.12547

**Published:** 2018-03-13

**Authors:** Catherine L. King, Maria Y. K. Chow, Kerrie E. Wiley, Julie Leask

**Affiliations:** ^1^ National Centre for Immunisation Research and Surveillance The Children's Hospital at Westmead Westmead NSW Australia; ^2^ Discipline of Child and Adolescent Health Sydney Medical School The University of Sydney The Children's Hospital at Westmead Clinical School Westmead NSW Australia; ^3^ Western Clinical School Sydney Medical School Level 2, Clinical Sciences Corridor C24 ‐ Westmead Hospital The University of Sydney Westmead NSW Australia; ^4^ School of Public Health The University of Sydney Sydney NSW Australia; ^5^ Faculty of Nursing and Midwifery The University of Sydney Camperdown NSW Australia

**Keywords:** Australia, communication, influenza, pandemic, parents, trust

## Abstract

**Background:**

Effective public health messaging is essential in both the planning phase and duration of a pandemic.

**Objectives:**

This study aimed to gain an understanding of parental information seeking, trusted sources and needs in relation to pandemic influenza A 2009 (pH1N1) to inform future policy planning and resource development.

**Patients/Methods:**

We conducted a mixed methods study; parents from 16 childcare centres in Sydney, Australia, were surveyed between 16 November and 9 December 2009, and interviews were conducted with participants from six childcare centres between June 2009 and May 2011.

**Results:**

From 972 surveys distributed, 431 were completed; a response rate of 44%. Most parents (90%) reported that doctors were “trusted a lot” as a source of influenza information, followed by nurses (59%), government (56%) and childcare centres (52%). Less trusted sources included media (7% selected “trusted a lot”), antivaccination groups (6%) and celebrities (1%). Parents identified a range of key search terms for influenza infection and vaccine. From 42 in‐depth interviews, key themes were as follows: “Action trigger,” “In an emergency, think Emergency,” “Fright to hype” and “Dr Google and beyond.” Parents relied heavily on media messages, but cynicism emerged when the pandemic was milder than expected. Parents viewed a range of information sources as trustworthy, including doctors, authoritative hospital or government websites, and childcare centres and schools.

**Conclusions:**

A user‐centred orientation is vital for pandemic communications including tailored information provision, via trusted sources based on what parents want to know and how they can find it.

## 1 BACKGROUND

The 2009 influenza A H1N1 pandemic began with initial cases in North America in April 2009. By mid‐June 2009^1^ cases had been reported in 74 countries and the World Health Organisation formally declared a pandemic on 11 June 2009.^2^ There were initially a number of unknowns about the pandemic H1N1 (pH1N1) virus including its potential virulence and transmissibility.^3^, ^4^ Influenza vaccination can be an effective public health measure to help prevent influenza disease and associated complications,^5^, ^6^ but at the peak of the pandemic in Australia in July 2009,^7^ pH1N1 vaccines were not yet available. A monovalent H1N1 vaccine was registered in Australia in September 2009 and freely available for those aged 10 years and above^8^; this was later extended in December 2009 to include children aged 6 months to 9 years.^9^ There were approximately 37 000 laboratory‐confirmed cases of pH1N1, and 5000 hospitalisations and 191 deaths due to pH1N1 in Australia in 2009.^7^ The median age of those who died was much lower than in preceding influenza seasons (53 years, rather than 83 years).^10^ An international systematic review revealed a higher pH1N1 attack rate in children compared to older adults who had some immunity from previous exposure.^11^ More than 100 children were hospitalised in Australia during the pandemic period and 11 children died.^12^

Effective communication and public health messaging is a key component in both the planning phase for a health emergency such as a pandemic and during the emergency itself.^13^, ^14^ Public cooperation during a pandemic is essential to minimise disease spread, ensure compliance and support for hygiene and social distancing measures and vaccination efforts, and avoid unnecessary overload on the health system.^15^, ^16^ As a pandemic progresses, messages may need to be modified according to the changing context.^15^ The pandemic communication strategy in Australia involved communication of hygiene and social distancing measures in May 2009, information about the availability of the pH1N1 vaccine in September 2009, availability of the pH1N1 vaccine for children in December 2009 and further vaccination information in March 2010.^7^

Public health messages are received and interpreted contextually and according to individual experiences.^15^, ^17^ Parents are the key enablers of whether or not their children comply with public health measures, including vaccination, and trust plays a key role in decision‐making in both pandemic^16^, ^18^, ^19^ and non‐pandemic periods.^20^ It is therefore important to know who and what parents trust to provide information about influenza and influenza vaccine in a pandemic context.

Thus, this study aimed to explore what information sources parents trusted and used to obtain information about pH1N1, during both the acute and post‐pandemic phase. Further, it examined how parents searched for information on influenza infection and influenza vaccine. An understanding of parental information needs and searching preferences could provide valuable insights to inform future pandemic planning and information campaigns.

## 2 METHODS

This study, conducted in Sydney, Australia, was part of a broader study examining the health, social and economic impacts of vaccinating children attending childcare against influenza.

We used mixed methods—a quantitative survey and qualitative semi‐structured interviews with parents of children aged 6 months to 5 years. Use of mixed methods combines the strengths of both quantitative and qualitative approaches and allows for a more robust exploration of an issue.^21^

The timing of the survey distribution and interviews in relation to external pH1N1 events is contained in Table 1.

**Table 1:** Study context

Date	Context	Interview/Survey timing
April 2009	H1N1 emergence reported in North America	–
June 2009	World Health Organization declared influenza pandemic first Australian pH1N1 death	Pilot interviews (n = 5)
September 2009	Free monovalent pH1N1 influenza vaccine available for those aged 10 y and above	–
November 2009	–	Interviews (n = 2) Survey data collection
December 2009	Free monovalent pH1N1 vaccine available for children aged 6 mo to 9 y	Interviews (n = 2) Survey data collection
March 2010	Adverse events reported in children following administration of seasonal influenza vaccine containing pH1N1	Interviews (n = 8)
23 April 2010	Chief Medical Officer suspended influenza vaccine for children under 5 y of age	–
June 2010	–	Interviews (n = 6)
2 July 2010	Initial regulator (TGA) report released	Interviews (n = 3)
30 July 2010	Non‐affected influenza vaccines for children available again	–
24 September 2010	Updated report released by TGA	–
October 2010	Report providing an overview of the incident released by TGA	Interviews (n = 5)
November 2010	–	Interviews (n = 3)
May 2011	Final TGA investigation findings released (after interviews finished)	Interviews (n = 8)

### 2.1 Quantitative survey

To inform the questions for the quantitative survey, the first author (CK) conducted five pilot qualitative interviews between 18 and 25 June 2009 at a Sydney metropolitan childcare centre. Parents were asked whether they had sufficient information about pandemic influenza, where they had obtained the majority of their information, and who or what they trusted to give them reliable information about pandemic influenza. The term “swine flu” was specifically used in interview questions as this closely mirrored the terminology commonly used in the media at the time.

Responses from these interviews formed the basis for quantitative questions on information sources and trust. These questions were designed by three authors (CK, MC and JL). Parents were asked to rate their level of trust in nine information sources using a Likert scale. Parents also had the option to use free text responses to nominate any other trusted sources, and to indicate how they would undertake an Internet search for information on influenza infection and influenza vaccine. Wording was kept general so parents could answer for either seasonal or pandemic influenza (swine flu) as, by November 2009, pH1N1 was the predominant circulating strain in Australia.^22^ Full methods for the questionnaire sampling, distribution and collation have been previously described.^23^

The quantitative survey was conducted in 16 Sydney metropolitan childcare centres across regions of varied socio‐economic status between 16 November and 9 December 2009. Parents had the option to complete either a written or web‐based questionnaire.

The resulting questionnaire data were analysed by all authors (CK, MC, KW and JL). Frequencies for the trusted sources were calculated and graphed. A chi‐square test analysis was undertaken using SPSS version 24 to examine demographic variables and trust in information sources. NVIVO 10 software was used to determine the frequency of individual search terms used.

### 2.2 Qualitative interviews

The first author (CK) conducted an additional 37 semi‐structured interviews between 24 November 2009 and 24 May 2011, across an additional five childcare centres. Interviews continued until theoretical saturation of the topics was reached. Four centres were utilised for both survey distribution and interviews.

Each interview was recorded with participant consent and then transcribed word‐for‐word. Qualitative research software, NVIVO 10, was used by the first author (CK) to assign codes to both the pilot and subsequent interviews. Using a thematic analysis approach informed by elements of grounded theory,^24^ interviews were coded initially by the first author using a line‐by‐line methodology. Initial analysis was completed soon after each set of interviews. Subsequent coding phases compared initially coded items with new interview data and examined the relationships between emerging themes. All co‐authors analysed a subset of the interviews to compare, refine and finalise themes.

## 3 RESULTS

### 3.1 Quantitative survey

There were 431 completed surveys from 972 distributed, a response rate of 44%. Demographic details of participants have been previously reported in full and found to be more highly educated than the general population (in which the rate for a university qualification is 24%)^23^; in contrast, participants in our study were predominantly highly educated (postgraduate qualification 48%; undergraduate 27%) mothers (90%) aged between 31 and 40 years (70%). A chi‐squared analysis revealed no significant associations between demographic variables and information sources, with the one exception of parental education level and trust in natural therapists (defined as Complementary and Alternative Medicine [CAM] practitioners, which include naturopaths, homeopaths and herbal medicine practitioners; χ^2^ = 5.58, *df *= 1, *P *=* *.02). Parents with a university education were less likely to trust their natural therapist, compared with parents without a university education (61% vs 76%, OR 0.5, 95% CI 0.3‐0.9).

**Figure 1:**
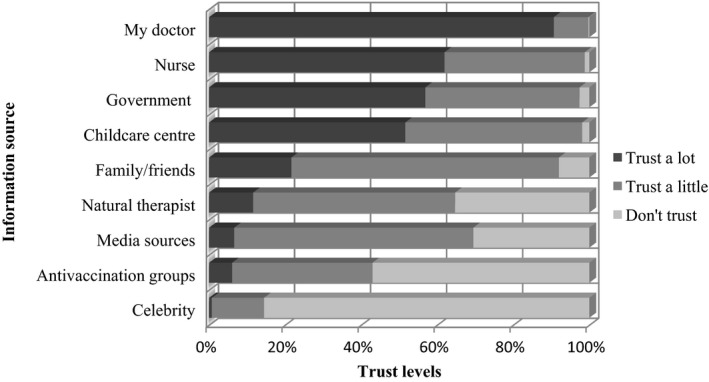
Parental trust levels for information sources

Parents reported that people they “trusted a lot” with regard to influenza information included their doctor (90%), nurses (59%), government (56%) and childcare centres (52%). The media was only “trusted a lot” by 7% of participants. Celebrities and antivaccination groups were not well trusted. These results are more fully explored in Figure 1.

In addition, 79/431 (18%) participants provided free text responses noting other sources of trusted information. The most frequently mentioned additional trusted sources were research/researchers (29%), doctors, especially paediatricians (14%), early childhood health clinics (12%) and hospitals (8%).

In relation to parental search terms, 384/431 (89%) participants provided responses on how they would use a search engine to find information on “influenza infection” and 366/431 (85%) participants provided responses on how they would search for information on “influenza vaccine.” The 10 most commonly used individual parental search terms for “influenza infection” and “influenza vaccine” are available in Tables 2 and 3, respectively.

**Table 2:** Ten most frequently reported individual search terms for “influenza infection” from the quantitative survey

Word	Frequency	% Overall word count
Flu	352	37.5
Influenza	82	8.7
Symptoms	59	6.3
Vaccine	53	5.6
Infection	47	5.0
Children	35	3.7
Google	22	2.3
Vaccination	15	1.6
Virus	13	1.4
Immunisation	11	1.2

**Table 3:** Ten most frequently reported individual search terms for “influenza vaccine” from the quantitative survey

Word	Frequency	% Overall word count
Flu	319	31.9
Vaccine	201	20.1
Influenza	58	5.79
Vaccination	37	3.7
Children	35	3.5
Immunisation	33	3.3
Shot	16	1.6
Effects	13	1.3
Side	13	1.3
Google	12	1.2

Of note is the strong preferential use of “flu” rather than “influenza” by parents, despite the potential priming effect of using “influenza” in the questions. The main synonyms mentioned by parents for vaccine included “shot,” “jab,” “needle” and “inoculation.” Also interesting was the use by some parents of geographic limiters, for example, Australia or Sydney, suggesting a preference by some for local information. When listing terms for “influenza infection,” a few parents used the colloquial term “bug” and one parent noted “I would not Google this, who has the time?”. When listing terms for “influenza vaccine,” there were a few sophisticated responses including a search for “official trials” and “put the name of the vaccine [and] search in PubMed.”

### 3.2 Qualitative interviews

Including the pilot interviews, 42 interviews were conducted with parents (41 mothers and one father). The majority of the parents were between 31 and 40 years of age (n = 33), Australian born (n = 26), university educated (n = 28) and working outside the home (n = 28). Demographic details have been published previously.^25^ To preserve confidentiality, pseudonyms were assigned to all participants for reporting purposes. Key emergent themes in relation to parental experiences of the pandemic are discussed below.

#### 3.2.1 Action trigger

The majority of parents thought they had not received enough information about the pandemic. Chief among parents’ expressed information needs was knowing when to seek medical attention if their child experienced influenza symptoms. Parents expressed a great sense of urgency about needing to know what signs and symptoms of illness should prompt action and they appeared frustrated at the lack of information provided on this topic.

The majority of parents recalled receiving preventive health messages about the pandemic, including instructions on cough etiquette, hand hygiene and use of social distancing measures. However, as described by Kim in June 2010,

take precautions, cover your face… then there wasn't anything else after that… all this scare information to start with, but we haven't really got anything else that we can run with.

Related to this was an expressed need to be able to differentiate between pandemic influenza and seasonal influenza symptoms. Many parents wanted a checklist to guide them. As articulated by Martha early in the pandemic in June 2009,

What is the trigger that sends me off to the Emergency Department?… That's the crucial information… On the news… they don't give a clear list of ‘if this happens do this’ or ‘this is what you look out for’.

#### 3.2.2 In an emergency, think Emergency

Parents trusted general practitioners (GPs), but in the pandemic context—a public health emergency—many parents expressed a preference for attending hospital Emergency Departments. Hospitals were accessible 24 hours a day, in contrast to GP services which some parents described as being difficult to access in a timely manner. Parents also perceived hospitals to be better prepared and equipped to deal with the pandemic, with some parents feeling that GPs lacked pandemic‐specific knowledge. As detailed by Juliana in October 2010,

I'd just go to the hospital, because I don't believe the GPs are really prepared for that… they don't have it in their mind. I have never ever heard from them about swine flu.

This perception that hospitals were the best place to take a child with pH1N1 caused one parent to deliberately avoid taking her young child to hospital during a prolonged vomiting illness as she was concerned the child would be exposed to pH1N1.

#### 3.2.3 Fright to hype

Parents reported high media usage and reliance throughout the pandemic, due to the rapidly changing situation. Parents interviewed in the initial phases of the pandemic were very concerned about the virulence of the virus and its possible adverse impact on their family in the form of both long‐ and short‐term health outcomes. They were also concerned about the practical implications of caring for an unwell child and the potential impact of quarantine, including on paid employment, and were troubled by the lack of a vaccine. These fears were much less prevalent in parents interviewed later in the pandemic as their experience of pH1N1 did not mirror the media messages they had initially received. Yasmin, in October 2010, described being

terrified at first, but then at that time they didn't have any kind of vaccinations against it, or they were working on it, but nothing was concrete, but then as time went on, it just seemed to fizzle out, and … as the months went by, it didn't seem to be turning out as they expected it to, so yes, it didn't worry me that much after that.

Indeed, the mildness of the pandemic compared to initial predictions, the intensity of the media coverage and the lack of exposure to pH1N1 by them or their family or friends led to a sense of disillusionment and loss of trust in many parents. As recalled by Diane in May 2011,

there was all this hype and panic about it, but then it was like ‘oh we don't actually know how bad it is, but let's freak out anyway’.

A small number of parents expressed being either unconcerned or disinterested in the H1N1 pandemic. This was often proximity related; for example, a lack of concern was linked to a lack of personal or immediate contact exposure to pH1N1.

#### 3.2.4 Dr Google and beyond

While the survey indicated that the mass media ranked low on the list of trusted influences, the interviews reflected the degree to which the media underscored parental knowledge. Media reliance was considered necessary, if untrustworthy. Parents also identified a variety of important additional sources for pH1N1 information. Parents trusted information provided by schools, childcare centres, hospitals and doctors. Hospitals, family GPs and childcare centre staff were seen as knowledgeable about local circulating virus strains. Internet searching was viewed as a convenient option, available at the point of information need. Parents were discerning about information quality and expressed a preference for reliable, authoritative websites. Hospital and government websites, as well as agencies such as the World Health Organization, Centers for Disease Control and Prevention and the Australian Medical Association, were specifically mentioned as trusted. As stated by Paula in May 2011,

I trust… government health websites. I wouldn't just look at any old website.

One parent described in detail how the use of a trusted source, a doctor, on a popular sports programme allayed her concerns about pH1N1.

## 4 DISCUSSION

This mixed methods study provides insights into Australian parents’ trust, perceptions, information needs and information source preferences as the H1N1 pandemic evolved. A strength of the study is the utilisation of mixed methods. The initial qualitative interviews directly informed the development of the questionnaire, and then, the subsequent interviews provided a richer understanding of some aspects of the quantitative responses. Continuing qualitative interviews into the post‐pandemic period also allowed us to explore parental views over time.

This study reveals that parents had unmet information needs in relation to the pandemic, particularly in regard to differentiating pH1N1 from other respiratory viruses and knowing when to seek medical assistance. While there are difficulties differentiating pH1N1 from other respiratory viruses,^26^ parents needed to be informed of this. Clear information, in the form of a checklist matching child symptoms to parental actions, may have assisted parents in decision‐making about presentation to health services. Another Australian study in August and September 2009 found that only 14.5% of respondents could correctly answer questions concerning influenza viral spread, infection control and symptoms, suggesting that either information dissemination was lacking or that messages were not sufficiently getting through to the public.^27^

The Australian government did provide information at key points throughout the pandemic period,^7^ yet the overwhelming perception of parents in this study was that, apart from initial messages about hygiene measures, little other information was forthcoming. Another Australian study conducted early in the pandemic in May 2009 revealed that 44% of those interviewed felt they did not have enough information about the pandemic.^28^ An analysis of television coverage of the pandemic in Australia between 25 April and 9 October 2009 found that information provided included discussion of the potential seriousness of pH1N1, the changing alert level and infection rates. There was very little, however, contextualised information about what the risk may mean for an individual or what they should be actively doing (apart from infection control measures such as handwashing).^29^ This supports the findings from our qualitative study where parents recalled receipt of infection control messages but then little else.

Our study found that parents increasingly reported feeling that pandemic risks had been exaggerated and/or sensationalised in the media. This finding is supported by studies in both Australian and international contexts.^30^, ^31^, ^32^ This increased cynicism could be due to the eventual mildness of the pandemic and therefore the perception of conflicting messages, in combination with the lack of personal experience with or exposure to pH1N1.^18^ A study conducted in Sydney, Australia, between 5 September and 3 October 2009 found that less than 20% of those interviewed believed they were at high risk of contracting pH1N1. Further, 69% of these respondents had no direct or indirect experience (via family or friends) of pH1N1 illness.^33^ Others have suggested the intense focus on basic infection control measures such as hand hygiene rather than more complex measures may have resulted in a perceived lack of seriousness.^27^

Despite the increased scepticism among parents about the media reportage of the pandemic, the quantitative responses in our study showed that, in common with other studies in the Australian context,^34^, ^35^, ^36^ parents continued to value and trust doctors for healthcare information. The qualitative responses in our study allowed for a more nuanced understanding of this issue and revealed that while GPs are trusted, in a pandemic context they were seen as more difficult to access and perceived as being less prepared to deal with pandemic influenza than hospitals. While some parents in the study were passive information seekers, others turned to the Internet and actively sought pandemic information. These parents displayed a preference for information from authoritative websites or sources such as hospitals, doctors and government.

### 4.1 Limitations

Limitations of this study include the questionnaire response rate of 44%. As previously described, this is within the response rate range of other parental vaccine surveys.^23^ A further potential limitation is that the questionnaire did not specify seasonal or pH1N1 influenza. While this was a deliberate choice given that pH1N1 was the dominant strain by the time of the questionnaires, it may have resulted in some parental confusion, and it is difficult to know whether parents were definitively answering for seasonal or pH1N1 influenza.

Another limitation was limited generalisability due to selection bias. Many of the participants in both the questionnaire and interviews were highly educated, which may have influenced responses, including the specificity of the search methods they used. Social desirability bias in which idealised answers are provided could also have impacted on the results. This is less likely in a self‐administered questionnaire compared to a researcher‐administered questionnaire.^37^

### 4.2 Recommendations for public health

The results of this study have implications for pandemic preparedness; in particular, parental preference for presentation to hospital rather than GPs has the potential to overwhelm hospital resources in the event of a more severe pandemic. The media remains an important mode of dissemination of regular information throughout the duration of a pandemic as suggested in our participant's reliance on it, despite a stated view of its lack of trustworthiness. This finding of the centrality of the media's role concurs with emergency situation literature and post‐pandemic evaluations of communication efforts. These suggest the need for clear, carefully crafted and tailored messages with a key role for health professionals.^38^, ^39^

Trusted sources such as doctors, government health department representatives and researchers could be utilised in both traditional media spaces and in non‐conventional settings such as on popular programs. Providing and promoting a hotline staffed by trusted sources such as doctors and nurses could assist in disseminating advice to guide appropriate presentation at Emergency Departments. In addition, factsheets developed by trusted sources (including hospitals) could be available physically in GP surgeries, hospitals, schools and childcare centres, and on websites.

Resources for use during a pandemic should take into account the preferred search terminology expressed by parents, for example, the use of the more informal “flu” rather than “influenza.” To optimise search engine retrieval, metadata underpinning resources could use this as a variant term so that resources can be effectively located by parental Internet searches.^40^

## 5 CONCLUSION

Understanding and considering the range of views, information needs, and preferences for searching and sources expressed by parents during the pandemic period provides useful context for developing tailored information materials and messages. Using and further promoting trusted sources via the media, as well as using existing trusted sources such as childcare centres and schools, could assist in disseminating public health messages in the event of future pandemics.

## COMPETING INTERESTS

The authors have no competing interests.

## ETHICAL APPROVAL

The study was granted ethics approval from the Human Research Ethics Committee of The Children's Hospital at Westmead, Australia. Informed consent was obtained for participation in the study.
